# Antineutrophil cytoplasmic antibody profiles differ according to type of primary sclerosing cholangitis and autoimmune hepatitis

**DOI:** 10.6061/clinics/2021/e2228

**Published:** 2021-02-01

**Authors:** Juliana Goldbaum Crescente, Alessandra Dellavance, Marcio Augusto Diniz, Flair Jose Carrilho, Luis Eduardo Coelho de Andrade, Eduardo Luiz Rachid Cançado

**Affiliations:** I Departamento de Gastroenterologia, Faculdade de Medicina FMUSP, Universidade de Sao Paulo, Sao Paulo, SP, BR; II Laboratorio de Investigacao Medica (LIM 06), Instituto de Medicina Tropical, Universidade de Sao Paulo, Sao Paulo, SP, BR; III Disciplina de Reumatologia, Escola Paulista de Medicina, Universidade Federal de Sao Paulo, Sao Paulo, SP, BR; IV Divisao de Pesquisa e Desenvolvimento, Laboratorios Fleury Medicina e Saude, Sao Paulo, SP, BR; V Biostatistics and Bioinformatics Research Center, Samuel Oschin Comprehensive Cancer Institute, Cedars Sinai Medical Center, Los Angeles, CA, USA

**Keywords:** Antibodies, Antineutrophil Cytoplasmic, Autoimmune Hepatitis, Cholangitis Sclerosis, Inflammatory Bowel Diseases

## Abstract

**OBJECTIVES:**

To determine the frequency of the antineutrophil cytoplasmic antibodies (ANCA), antiproteinase-3 and antimyeloperoxidase, in primary sclerosing cholangitis (PSC) with or without inflammatory bowel disease (IBD+ or IBD-) and in different types of autoimmune hepatitis (AIH). Additionally, to verify the agreement between ANCA patterns by indirect immunofluorescence and their antigenic specificities by ELISA.

**METHODS:**

For this study, 249 patients were enrolled (42 PSC/IBD+; 33 PSC/IBD-; 31 AIH type-1; 30 AIH type-2; 31 AIH type-3; 52 primary biliary cirrhosis; 30 healthy controls) whose serum samples were tested for ANCA autoantibodies.

**RESULTS:**

There were fewer female subjects in the PSC/IBD- group (*p*=0.034). Atypical perinuclear-ANCA was detected more frequently in PSC/IBD+ patients than in PSC/IBD- patients (*p*=0.005), and was significantly more frequent in type-1 (*p*<0.001) and type-3 AIH (*p*=0.012) than in type-2 AIH. Proteinase-3-ANCA was detected in 25 samples (only one with cytoplasmic-ANCA pattern), and more frequently in PSC/IBD+ than in PSC/IBD- patients (*p*=0.025). Myeloperoxidase-ANCA was identified in eight samples (none with the perinuclear-ANCA pattern). Among the 62 reactive samples for atypical perinuclear-ANCA, 13 had antigenic specific reactions for proteinase-3 and myeloperoxidase.

**CONCLUSIONS:**

PSC/IBD+ differed from PSC/IBD- in terms of sex and proteinase 3-ANCA and atypical perinuclear-ANCA reactivity, the latter of which was more frequently detected in type-1 and type-3 AIH than in type-2 AIH. There was no agreement between ANCA patterns and antigenic specificities in IBD and autoimmune liver diseases, which reinforces the need for proteinase-3 and myeloperoxidase antibody testing.

## INTRODUCTION

Antineutrophil cytoplasmic antibodies with atypical perinuclear patterns (A-ANCA) are frequently found in inflammatory bowel diseases (IBD), particularly in ulcerative colitis, primary sclerosing cholangitis (PSC), and autoimmune hepatitis (AIH) (1,2). Since PSC and IBD are closely associated, it is not clear whether A-ANCA reactivity is more related to IBD or PSC. Few studies have addressed the frequency distribution of this marker in IBD-associated (PSC/IBD+) and un-associated PSC (PSC/IBD-).

Unlike the cytoplasmic (C-ANCA) and perinuclear-ANCA (P-ANCA) patterns, which have a specificity for proteinase-3 (PR3-ANCA) and myeloperoxidase (MPO-ANCA), respectively, the A-ANCA pattern does not have a definite target antigen. Inconsistent results have been found when using neutrophil substrates fixed in ethanol and formalin or treated with deoxyribonuclease in the absence of a concomitant reactivity against PR3 and MPO enzymes by antigen-specific immunoassays (3,4). Due to a lack of international consensus on the standardization of different ANCA patterns, the specific techniques used for detecting A-ANCA in specialized laboratories are decided based on experience (3,4). Additionally, the reactivity of A-ANCA in IBD is difficult to assess due to the concomitant reactivity of PR3-ANCA (5,6).

In AIH, the A-ANCA pattern was more often reactive in AIH type-1 than AIH type-2, and ANCA reactivity has not been evaluated in AIH type-3, the main markers of which are anti-soluble liver antigen/liver-pancreas antibodies (anti-SLA/LP) (7).

The aim of this study was to evaluate the frequency of ANCAs using indirect immunofluorescence (IIF) and the frequency of PR3-ANCA and MPO-ANCA specifically using ELISA for PSC/IBD+, PSC/IBD-, different types of AIH, and primary biliary cholangitis (PBC). A second objective was to determine the agreement between different ANCA patterns by IIF and PR3-ANCA and MPO-ANCA reactivity determined by ELISA.

## METHODS

This cross-sectional study was comprised of 249 patients. Of them, 42 had PSC/IBD+ (41 of which had ulcerative colitis) and 33 had PSC/IBD-. Additionally, 92 had one of three types of AIH, 52 had PBC, and 30 were healthy controls. At the time of blood sample collection, the median age of the patients was 34.6 years for PSC/IBD+, 31.3 years for PSC/IBD-, 25.8 years for AIH-1, 20.6 years for AIH-2, 37.2 years for AIH-3, 51.0 years for PBC, and 40.8 years for the healthy controls.

PSC was diagnosed based on increased levels of alkaline phosphatase with cholangiographic changes or the presence of concentric periductal fibrosis on histological evaluation. To diagnose ulcerative colitis, colonoscopic examinations were assessed for compatible macroscopic and/or microscopic changes. The diagnosis of Crohn's disease was based on a set of alterations found in colonoscopy, histopathology, small bowel radiography, abdominal tomography, and/or magnetic resonance imaging. In PSC/IBD- patients, a colonoscopy was always performed to rule out the presence of IBD and, in 90% of cases, was accompanied by a histological confirmation of the absence of microscopic colonic involvement.

Initial serum samples or those collected during episodes of AIH relapse were tested to avoid using samples after a long period of corticoid and immunosuppressive treatment. Serum samples were stored at -20°C after performing a routine test to detect liver disease autoantibodies. In the case of patients with PSC/IBD+, this requirement was not always adhered to because many of the patients frequently received an immunosuppressive regimen in order to treat the IBD. Antismooth muscle (ASMA), antiliver kidney microsome type 1 (anti-LKM1), and antimitochondrial antibody testing was performed according to international guidelines on unfixed 4-μm thick sections of rat stomach, liver and kidney (8). Sera were diluted for screening in PBS/Tween 20 at dilutions of 1/40 and 1/80.

The International AIH Group recommendations were followed for the diagnosis of AIH, and only patients with a confirmed diagnosis were included (9). AIH-1 was defined as ASMA with reactivity against muscle fibers of the stomach, vessels, glomeruli, and renal tubule pericellular fibrils (8). All patients with AIH-2 had reactivity to anti-LKM1. AIH-3 was characterized by anti-SLA/LP antibodies and the absence of ASMA and anti-LKM-1 antibody reactivity. For all the subtypes, antinuclear antibodies may or may not have been present. Anti-SLA/LP antibodies were detected using anti-SLA/LP ELISA (Immunoglobulin G [IgG]) (Euroimmun, Lübeck, Germany) and/or by QUANTA lite^TM^ IgG SLA (INOVA Diagnostics Inc., San Diego, CA, USA).

For the diagnosis of PBC, patients had to have biochemical and/or histological cholestasis with simultaneous reactivity to antimitochondrial antibodies, evidenced by IIF, and/or to enzymes of the 2-oxoacid dehydrogenase complex, detected by ELISA (ORG 516 AMA-M2, ORGENTEC Diagnostika GmbH, Mainz Germany) commercial assay or immunoblotting (10). Patients with overlapping AIH syndromes were not included.

The IIF tests for ANCA reactivity were performed according to the manufacturer's recommendations (NOVA Lite^®^ ANCA, INOVA Diagnostics, San Diego, USA). Granular cytoplasmic reactivity observed on ethanol-fixed neutrophils was designated C-ANCA. The samples with perinuclear reactivity were re-tested in formalin-fixed neutrophils. Samples that changed from the perinuclear pattern to cytoplasmic were classified as P-ANCA and those that continued to have perinuclear reactivity were designated A-ANCA. Reactive samples with a fluorescent pattern that was not negative, possibly due to the interference of a concomitant antinuclear antibody reactivity, and did not meet the qualifications of any of the other ANCA categories were classified as inconclusive. All sera were also evaluated for the presence of antinuclear antibodies to clarify any interference with ANCA patterns using the commercial NOVA Lite^®^ HEp-2 ANA (INOVA Diagnostics, San Diego, CA, USA).

Sera samples were tested using an indirect ELISA to define the antigenic specificity of PR3-ANCA and MPO-ANCA. The commercial assays QUANTA lite^TM^ MPO IgG ELISA and QUANTA lite^TM^ PR3 IgG ELISA (INOVA Diagnostics, San Diego, CA, USA) were used according to the manufacturer's recommendations. For both ANCAs that were tested for using ELISA, a reactivity of <21 units or ≥21 units were identified as negative and positive, respectively. All patients signed a written informed consent form allowing for the preservation of serum samples collected for routine autoantibody detection testing to characterize autoimmune liver diseases. The scientific ethics committee of the Clinic Hospital of the University of Sao Paulo School of Medicine, and the Research Ethics Committee of Fleury approved the research protocol. The study followed the World Medical Association Declaration of Helsinki regarding ethical conduct involving human beings.

The results were reported as the mean (±standard deviation) and median for quantitative variables and frequency (percentage) for qualitative variables. Prevalence estimates were followed by their respective 95% confidence intervals (CIs). Multiple comparisons were made using Fisher's exact test, with Holm's correction when needed (11,12). The agreement between methods was analyzed using McNemar and kappa tests (13). McNemar tests the null hypothesis of agreement by comparing the marginal percentages of positive and negative results. Statistically significant results indicated that the percentage of positive/negative IIF results and antigenic specificities by ELISA were different. The kappa tests the null hypothesis of non-agreement by comparing the observed agreement (positive plus negative results) and the expected agreement occurring by chance. Statistically significant results indicated that the agreement between IIF and antigenic specificities by ELISA was not the result of chance. The results were considered statistically significant for *p-*values <5%. All calculations were performed using the program R, version 3.1.2 (14).

## RESULTS

The gender distribution was as follows: 29 male patients in PSC/IBD+ (69.0%) and 14 in PSC/IBD- (42.4%). In the other groups, most of the participants were women (AIH-1 [74.2%], AIH-2 [86.2%], AIH-3 [100%], PBC [85.5%], and healthy controls [70.0%]). The most significant difference was the higher prevalence of women in the PSC/IBD- group than in the PSC/IBD+ group (57.6% *vs.* 31.0%, respectively, *p*=0.034). Indeed, there were significantly more women in all the other study groups, with significantly higher rates in PBC than in PSC/IBD- (86.5% *vs.* 57.6%, respectively, *p*=0.004) and in AIH-3 than in AIH-1 (100% *vs.* 74.2%, respectively, *p*=0.0015).

A-ANCA was the most frequently detected pattern in the study, identified in 24.9% of the 249 samples tested and found more significantly in PSC/IBD+ than in PSC/IBD- (*p*=0.005). There was no significant difference in the frequency of A-ANCA between PSC/IBD- and PBC (*p*=0.56). However, there was a significant difference in the presence of A-ANCA among the subtypes of AIH (*p*<0.001). The A-ANCA pattern was seen more frequently in AIH-1 (*p*<0.001) and AIH-3 (*p*=0.012) than in AIH-2. There was no difference between the AIH-1 and AIH-3 groups (*p*=0.434). Table 1 shows the frequency of all the patterns.

Regardless of the patterns, overall ANCA reactivity was significantly more frequent in patients with PSC and AIH, with no significant difference between them (*p*=1), and was more frequent in the PSC/IBD+ group than in the PSC/IBD- group (*p*=0.037). Among the AIH subtypes, there was a significant difference between AIH-1 and AIH-2 (*p*=0.011) and between AIH-3 and AIH-2 (*p*=0.024). However, there were no significant differences between PSC/IBD+ and AIH-1 (*p*=1), between PSC/IBD+ and HAI-3 (*p*=0.746), between AIH-1 and AIH-3 (*p*=1), or between PBC and PSC/IBD- (*p*=1).

Twenty-five of the 249 samples tested positive for PR3-ANCA (10.0% [64% with reactivity above 37 units]), and it was more frequently detected in PSC/IBD+ than in PSC/IBD- patients (*p*=0.025). Eight of 249 samples were reactive to MPO-ANCA (3.2%, [50% with reactivity above 37 units]). The overall ELISA results for PR3-ANCA and MPO-ANCA showed more frequent detection in PSC compared to the other groups. These results are displayed in Table 2.

If considering the true A-ANCA pattern as being found only in samples that showed A-ANCA reactivity by IIF, but were negative in both ELISA (A-ANCA IIF+/ELISA-), 49 of the 249 samples had these features (19.7%) (Table 3). The occurrence of this pattern was significantly more reactive in the PSC/IBD+ group than in the PSC/IBD- one (*p*=0.02) but not between the PSC/IBD- and PBC groups (*p*=1). However, there was a significant difference among the subtypes of AIH (*p*=0.001). The A-ANCA IIF+/ELISA- pattern was seen more frequently in AIH-1 than in AIH-2 (*p*=0.001) and more in AIH-3 than in AIH-2 (*p*=0.012). There was no significant difference between the AIH-1 and AIH-3 groups (*p*=0.426).

There was evidence of marginal agreement (*p*=0.388, McNemar test) between the presence of P-ANCA observed by IIF and MPO-ANCA observed by ELISA; however, there was no evidence of central agreement (kappa coefficient=-0022, *p*=0.713). Among the four positive samples for P-ANCA, none had confirmed MPO-ANCA reactivity, and among the eight positive samples for MPO-ANCA, none had reactivity for P-ANCA. This total agreement was derived from negative results since none of the positive results were concordant with both methods.

The McNemar test showed evidence of marginal disagreement (*p*<0.001) between the presence of C-ANCA observed by IIF and PR3-ANCA by ELISA. Moreover, there was no evidence of central agreement once the kappa test was 0.05 (*p*=0.18). For negative results for both tests, the agreement was 89.2%. In the three samples with C-ANCA reactivity, one was PR3-ANCA positive. Similarly, one out of 25 PR3-ANCA positive samples exhibited the expected C-ANCA pattern according to IIF. Therefore, for the diseases evaluated in this study, there was no evidence that the C-ANCA pattern observed on IIF was concordant with PR3-ANCA reactivity.

Thirteen of the 62 A-ANCA-identified patterns showed reactivity to at least one of the ELISA tests. Twelve samples were positive for PR3-ANCA, and one showed simultaneous positivity for MPO-ANCA and PR3-ANCA (Table 3).

## DISCUSSION

The fact that men are more often diagnosed with PSC and less often with AIH and PBC is a *fait accompli*; however, in this study, women were more often diagnosed with PSC/IBD-. This finding is in accordance with a recent publication made on behalf of the International PSC Study Group (15).

A noteworthy finding of the present study was the significant difference in A-ANCA reactivity between PSC/IBD+ and PSC/IBD-, which was not always documented in the few studies conducted on this subject. Some authors have obtained similar results, one describes a P-ANCA reactivity of 88% in patients with PSC/IBD+ and of 40% in PSC/IBD- (16). In another study, in which the P-ANCA pattern was examined on formaldehyde-fixed neutrophils, and reactivity was based on a 1/20 titration, the frequency of P-ANCA in PSC/IBD+ and PSC/IBD- was 60% and 25%, respectively (17).

Although all patients with PSC/IBD- systematically underwent colonoscopy, serial biopsy samples were not collected in all patients as recommended, regardless of the normal macroscopic appearance of the mucosa. Therefore, asymptomatic cases of IBD with colonic changes that could only be confirmed by microscopy may have been missed, though this likely would not have altered the results of the study. On the contrary, the higher reactivity of A-ANCA in IBD cases may have increased the difference between the groups.

In contrast, some studies have found similar reactivity in these two clinical situations of PSC with or without associated IBD. One of them used two different techniques (ELISA with neutrophils fixed on the plates and IIF on ethanol-fixed neutrophils) and found a higher frequency of ANCA and P-ANCA in patients with ulcerative colitis and PSC (even in patients without IBD), respectively, than in patients with other liver diseases (18). In another report with a very limited number of samples, six of the eight PSC/IBD- patients (75%) and 14 of the 21 PSC/IBD+ patients (66.6%) were positive on the ethanol- and formalin-fixed neutrophil assay (19). Analysis of a Scandinavian cohort of 241 patients with PSC (192 PSC/IBD+ patients) with ANCA reactivity defined only by neutrophils fixed in ethanol from 1/20 titrations found no significant difference between PSC/IBD+ and PSCI/IBD- patients (20). Surprisingly, P-ANCA reactivity was higher than 65% in all subgroups (ulcerative colitis, Crohn’s disease, indeterminate colitis, and IBD-).

That A-ANCA reactivity is increased in AIH-1 is well known. In the most characteristic study, an impressive 96% of A-ANCA reactivity by ELISA was reported using plates with fixed neutrophils. What was most striking in this study was that 92% of the positive samples were also reactive according to IIF on ethanol-fixed neutrophils, with reactivity from a 1/50 titration (21). The low frequency of A-ANCA in AIH-2 is consistent with previously published data (7). Nevertheless, the A-ANCA reactivity in patients with AIH-3 is new information. The lower frequency of A-ANCA in patients with AIH-2 reinforces the distinction between AIH-1 and AIH-2. The similar frequency of this marker in AIH-1 and AIH-3 is an additional common characteristic for both subtypes.

In the current study, the P-ANCA pattern was rare in all the groups studied. This is consistent with another previous study, which found that of the 58 patients with IBD and 10 controls studied, only three patients with ulcerative colitis had this marker (22). However, this study did not distinguish between patients with PSC/IBD+ and PSC/IBD-. In our study and in the previous one, ethanol- and formaldehyde-fixed neutrophils were used as substrates to identify ANCA patterns, while for others, including the ANCA’s International Consensuses, such a requirement was unnecessary (3,4). In the current study, the C-ANCA pattern was rarely observed, which is consistent with the results from a study by Terjung et al. (23), which demonstrate that C-ANCA and P-ANCA patterns are, in fact, infrequent in IBD and autoimmune liver diseases. In the present study, there was no agreement between the presence of C-ANCA and P-ANCA patterns and PR3-ANCA and MPO-ANCA reactivity, and vice-versa. Although the finding that IIF methods have high variability and poor performance are in line with a multicenter European study and the recent international revised consensus on ANCA tests in vasculitis (24,25), it is impossible not performing them in favor of ELISA immunoassays because there are no target antigens for A-ANCA. Moreover, ANCA reactivity against two or more antigens was detected in approximately 50% of patients with ulcerative colitis, which also could help explain this discrepancy in results (26).

Due to contradictions in the concept of ANCA patterns and titers, the lack of agreement of the substrate fixation forms, and the inconsistency between the results of IIF and ELISA techniques, the overall reactivity of ANCA by IIF was analyzed regardless of the patterns. Even so, all significant differences discussed above remained. This also occurred when restricting the definition of A-ANCA to serum samples that showed reactivity according to IIF and negativity on ELISA.

The only group that showed reactivity above 10% for PR3-ANCA was PSC, and when analyzing PSC subgroups, the frequency was significantly higher in the PSC/IBD+ group than in the PSC/IBD- group. The importance of PR3-ANCA reactivity in ulcerative colitis has been reported, despite the low frequency of C-ANCA observed in IBD. A 2008 study compared the performance of a flow cytometric immunoassay for the detection of PR3-ANCA and MPO-ANCA with different commercial and in-house assays and detected a frequency of 27% and 17%, respectively (5). However, the inclusion of eight of the 22 patients with the C-ANCA pattern and 14 with the P-ANCA pattern may have skewed the results. On the other hand, while Mahler et al. found high PR3-ANCA reactivity in 31.1% of adult patients with ulcerative colitis and 1.9% of patients with Crohn's disease using a chemiluminescence technique, by ELISA the reactivity was only 6% in ulcerative colitis and absent in Crohn's disease. In that study, besides the higher reactivity of PR3-ANCA in ulcerative colitis compared to Crohn's disease, the presence of these antibodies was related to greater disease severity (27), which is not consistent with other publications on the subject (28,29).

Previous authors have found similar frequencies. In one study, PR3-ANCA reactivity in PSC was observed in 38.5% of samples by chemiluminescence and in 23.4% by ELISA (74.6% associated with IBD) (6). Nevertheless, there were also no significant differences between PSC/IBD+ and PSC/IBD- patients. In fact, the frequency of PR3-ANCA in IBD varied substantially when compared using different methods (30).

It is remarkable that in a multicenter study conducted to improve the interpretation of ANCA-associated vasculitis, a threshold higher than 37 units for antibody levels was found to improve diagnosis with a 77.3% sensitivity, 99% specificity, 79.4% positive likelihood, and 23% negative likelihood with 95% CIs (31). In the present study, using the same commercial kit, 64% of positive PR3-ANCA samples, and 50% of positive MPO-ANCA samples exhibited reactivity over that threshold Therefore, in order to define measures of diagnostic accuracy for ANCA-associated vasculitis, it is essential to include liver and intestinal autoimmune diseases in control groups.

The results obtained from IIF and specific antigen immunoassays, such as ELISA or chemiluminescence, produce very discordant results regarding the detection of ANCA, PR3-ANCA, and MPO-ANCA, as previously mentioned. This is likely explained by differences in diagnostic criteria for liver diseases, forms of therapy at the time of blood collection, and certainly differences in conformational forms of the antigens in the assays. More uniform results were expected when using commercial tests for IIF and ELISA rather than in-house assays, but this did not occur. For these reasons, it is difficult to reach definite conclusions, and the results in one cohort of patients cannot be extrapolated to all others. Moreover, these differences can also be attributed to environmental factors and/or different genetic susceptibilities. In this sense, Hov et al. has highlighted the greater genetic susceptibility of patients with HLA B8 and DR3 alleles in patients with positive P-ANCA PSC compared to those with negative P-ANCA PSC (20). HLA testing was not performed in the present study, but in a previous analysis of the frequency of HLA alleles in Brazilian patients with PSC, HLA DR*13 was the most frequent allele of susceptibility (32).

## CONCLUSIONS

In terms of gender and A-ANCA and PR3-ANCA reactivity, PSC/IBD+ and PSC/IBD- had different features. The reactivity of these antibodies seems to be more closely associated with IBD than with PSC. Just as IBD with PSC appears to be a phenotypically different entity from IBD without PSC (33), PSC alone also appears to have different characteristics than PSC/IBD+. While patients with AIH-1 and AIH-3 had similar A-ANCA reactivity results, both were different from AIH-2. For IBD, PSC, and AIH, there was no agreement between the IIF results for ANCA patterns and the PR3-ANCA and MPO-ANCA ELISAs, which showed high levels of reactivity, as observed in ANCA-associated vasculitis.

## AUTHOR CONTRIBUTIONS

Crescente JG drafted the study, performed all laboratory tests, and collected clinical data from patients. Dellavance A conceived of the ANCA testing approach and analyzed the indirect immunofluorescence tests. Diniz MA performed all statistical analyses of the study. Carrilho FJ critically analyzed the results. Andrade LEC contributed to the immunofluorescence test analyses and supervised the study. Cancçado ELR conceived of the study idea, proofread the study, critically analyzed the results, and was also responsible for assisting patients at the Gastroenterology Service outpatient clinic.

## Figures and Tables

**Table 1:** Reactivity of all ANCA patterns by indirect immunofluorescence for different autoimmune liver diseases and healthy controls.

Groups (N)	p-ANCA	A-ANCA	c-ANCA	Ac-ANCA	Inconclusive	Total positive
	N (%)	N (%)	N (%)	N (%)	N (%)	N (%)
	95% CI	95% CI	95% CI	95% CI	95% CI	95% CI
PBC	1 (1.9)	8 (15.4%)	1 (1.9)	0 (0.0)	0 (0.0)	10 (19.2)
(52)	0-11.1	7.7-27.8	0-11.1	0-8.2	0-8.2	10.6-32.09
PSC	3 (4.0)	29 (38.7)	1 (1.3)	1 (1.3)	6 (8.0)	40 (53.3)
(75)	0.9-11.6	28.4-50.0	0-7.9	0-7.9	3.41-16.7	42.2-64.2
PSC/IBD+	3 (7.1)	22 (52.4)	1 (1.3)	1 (1.3)	2 (4.8)	29 (69.0)
(42)	1.8-19.7	37.7-66.6	0-13.4	0-13.4	0.46-16.7	53.9-81.0
PSC/IBD-	0 (0)	7 (21.2)	0 (0)	0 (0)	4 (12.1)	11 (33.3)
(33)	0-12.4	10.4-38.1	0-12.4	0-12.4	4.21-27.9	19.7-50.5
AIH	0 (0.0)	25 (27.2)	1 (1.1)	1 (1.1)	7 (7.6)	34 (37.0)
(92)	0-4.8	19.1-37.1	0-6.5	0-6.5	3.5-15.1	27.8-47.2
AIH-1	0 (0.0)	14 (45.2)	1 (3.2)	0 (0.0)	1 (3.2)	16 (51.6)
(31)	0-13.1	29.2-62.2	0 -17.6	0-13.1	0-17.6	34.8-68.0
AIH-2	0 (0.0)	1 (3.3)	0 (0.0)	1 (3.3)	1 (3.3)	3 (10.0)
(30)	0-13.5	0-18.1	0-13.5	0-18.1	0-18.1	2.7-26.4
AIH-3	0 (0.0)	10 (32.3)	0 (0.0)	0 (0.0)	5 (16.1)	15 (48.4)
(31)	0-13.1	18.5-50.0	0-13.1	0-13.1	6.6-33.1	32.0-65.0
Controls	0 (0.0)	0 (0.0)	0 (0.0)	0 (0.0)	1 (3.3)	1 (3.3)
(30)	0-13.5	0-13.5	0-13.5	0-13.5	0-18.1	0-18.1
Total (249)	4 (1.6%)	62 (24.9%)	3 (1.2%)	2 (0.8%)	14 (5.6%)	85 (34.1)

PBC, primary biliary cholangitis; PSC/IBD+, primary sclerosing cholangitis with inflammatory bowel diseases, PSC/IBD-, primary sclerosing cholangitis without inflammatory bowel diseases; AIH-1, autoimmune hepatitis type 1; AIH-2, autoimmune hepatitis type 2, AIH-3, autoimmune hepatitis type 3; ANCA, antineutrophil cytoplasmic antibodies; p-ANCA, perinuclear pattern; A-ANCA, atypical p-ANCA; c-ANCA, cytoplasmic pattern; Ac-ANCA, atypical c-ANCA.

**Table 2:** Detection of antimyeloperoxidase (MPO-ANCA) and antiproteinase-3 (PR3-ANCA) antibodies by ELISA in different autoimmune liver diseases and healthy controls.

Groups (N)	MPO-ANCA	Threshold^#a^	PR3-ANCA	Threshold^a^	MPO-ANCA+ PR3-ANCA
	N (%)	>37 U	N (%)	>37 U	N (%)
	95% CI	N (%)	95% CI	N (%)	95% CI
PBC	0 (0.0)	0	2 (3.8)	2	2 (3.8)
(52)	0-8.22	0	0.3-13.7	2	0.32-13.7
PSC	4 (5.3)	4 (5.3)	19 (25.3)	13 (17.3)	21 (28.0)
(75)	1.69-13.3		16.8-36.3		19.1-39.1
PSC/IBD+	3 (7.1)	3 (7.1)	15 (31.0)	9 (21.4)	16 (38.1)
(42)	1.8-19.7		22.9-50.9		24.97-53.22
PSC/IBD-	1 (3.0)	1 (3.0)	4 (12.1)	4 (12.1)	5 (15.2)
(33)	0-16.7		4.5-29.5		6.17-31.4
AIH	4 (4.3)	0	3 (3.3)	1 (1.0)	7 (8.7)
(92)	1.36-11	0	0.72-9.55		3.49-15.12
Type 1	2 (6.5)	0	1 (3.2)	0	3 (9.7)
(31)	0.7-21.8	0	0-17.6	0	2.56-25.69
Type 2	2 (6.7)	0	1 (3.3)	0	3 (10.0)
(30)	0.8-22.4	0	0-18.1	0	2.66-26.42
Type 3	0 (0.0)	0	1 (3.3)	1 (3.2)	1 (3.3)
(31)	0-13.1	0	0-17.6		0-17.6
Healthy controls	0 (0.0)	0	1 (3.3)	0	1 (3.3)
(30)	0-13.47	0	0-18.1	0	0-18.09
Total (249)	249	249	249	249	249
positive	8 (3.2)	4 (1.6)	25 (10.0)	16 (6.4)	31 (12.5)
negative	241 (96.8)	245 (98.4)	224 (90.0)	233 (93.6)	218 (87.5)

a) threshold >37 units: 99% specificity for diagnosing ANCA-associated vasculitis (31).

b) two patients showed simultaneous reactivity for both antibodies. One exhibited the Ap-ANCA pattern and the other the atypical c-ANCA by IIF.

PBC, primary biliary cholangitis; PSC/IBD+, primary sclerosing cholangitis with inflammatory bowel diseases; PSC/IBD-, primary sclerosing cholangitis without inflammatory bowel diseases; AIH, autoimmune hepatitis.

**Table 3:** Anti-myeloperoxidase (MPO-ANCA) and anti-proteinase-3 (PR3-ANCA) antibodies in positive samples for atypical p-ANCA in autoimmune liver diseases and in healthy controls.

Groups	A-ANCA+ and ELISA+^a^ N (%)	A-ANCA+ and ELISA- N (%)
	95% CI	95% CI
Primary biliary cholangitis (52)	1 (1.9)	7 (13.5)
	0.0-11.1	6.4-25.6
Primary sclerosing cholangitis (75)	10.0 (13.3)	19 (25.3)
	7.2-23.0	16.8-36.3
PSC/IBD+ (42)	7 (16.6)	15 (35.7)
	8.0-30.9	22.9-50.9
PSC/IBD- (33)	3 (9.1)	4 (12.1)
	2.4-24.3	4.2-27.9
Autoimmune hepatitis (92)	2 (2.2)	23 (25.0)
	0.1-8.1	17.2-34.8
Type 1 (31)	1 (3.2)	13 (41.9)
	0-17.6	26.4-59.3
Type 2 (30)	0 (0.0)	1 (3.3)
	0.0-13.5	0-18.1
Type 3 (31)	1 (3.2)	9 (29.0)
	0.0-17.6	15.9-46.8
Controls (30)	0 (0.0)	0 (0.0)
	0.0-13.5	0-13.5
Total (249)	13 (5.2)	49 (19.7)

a) ELISA-positive for at least one of the following: MPO-ANCA or PR3-ANCA.

A-ANCA, atypical antineutrophil cytoplasmic antibodies (ANCA); PSC/IBD+, primary sclerosing cholangitis associated with inflammatory bowel disease; PSC/IBD, primary sclerosing cholangitis not associated with inflammatory bowel disease.
